# Essential Oils as an Antifungal Alternative to Control Several Species of Fungi Isolated from *Musa paradisiaca*: Part II

**DOI:** 10.3390/microorganisms13112477

**Published:** 2025-10-29

**Authors:** Maritza D. Ruiz Medina, Jenny Ruales

**Affiliations:** Departamento de Ciencias de Alimentos y Biotecnología (DECAB), Escuela Politécnica Nacional (EPN), Quito 170143, Ecuador; jenny.ruales@epn.edu.ec

**Keywords:** essential oils, natural antifungals, postharvest diseases, antifungal alternative, postharvest management, banana

## Abstract

Essential oils (EOs) from oregano (*Origanum vulgare*), rosemary (*Salvia rosmarinus*), clove (*Syzygium aromaticum*), thyme (*Thymus vulgaris*), cinnamon (*Cinnamomum verum*), and basil (*Ocimum basilicum*) possess antifungal properties. This study aimed to evaluate their ability to inhibit the growth of fungi isolated from the rot of banana peel (*Musa paradisiaca*) to control or reduce fungal growth in bananas. The methodology involved preparing dilutions of EOs and inoculating them onto Potato Dextrose Agar (PDA) medium amended with chloramphenicol to prevent bacterial contamination. Fungal species, including *Trichoderma* spp., *Aspergillus* spp., *Penicillium* spp., and *Fusarium* spp., were isolated, purified, and characterized macroscopically and microscopically. Their growth was assessed ex vivo and the inhibition percentage was measured in vitro. The ex vivo analysis revealed that the severity of fungal infection, ranked from highest to lowest, was as follows: *Penicillium* spp., *Trichoderma* spp., *Fusarium* spp., and *Aspergillus* spp. The results showed that rosemary and basil oils did not inhibit fungal growth, whereas clove oil, cinnamon, and oregano were effective against the four tested fungi at 800, 400, and 200 ppm, respectively. These findings suggest that certain EOs, including clove, cinnamon, and oregano, have strong antifungal potential and could serve as eco-friendly alternatives to synthetic fungicides in banana postharvest disease management.

## 1. Introduction

Banana (*Musa paradisiaca*), a staple food in tropical regions and in many developing countries, plays an essential role as a pillar for the economic growth and social development of local communities, due to its ability to maintain consistent production throughout the year [1]. Ecuador’s banana production is vital for its economy and food security [2]. The main banana-producing provinces are Guayas, El Oro, and Los Ríos, with El Oro bananas notable for their quality, while Guayas and Los Ríos are the major producers [2,3].

In production, various factors, both external and internal, affect the quality of the fruit during the postharvest phase. External factors include environmental conditions such as relative humidity and temperature, and among internal factors, metabolic changes and the presence of fungal pathogens are highlighted [2,4].

It is important to emphasize that fungal pathogens, in particular diseases associated with *Fusarium* spp., *Penicillium* spp., *Trichoderma* spp., and *Aspergillus* spp., represent the main causes of banana wilt [5,6]. These diseases manifest as a reduction in the firmness of the superficial tissues in the areas of the rachis and the banana crown, accompanied by changes in leaf coloration and cracks at the base of the pseudo-stem [7]. This causes internal rot in the fingers of the banana, eventually resulting in crown rot and, in more severe cases, the death of the fruit [8,9].

Traditionally, these diseases have been managed using synthetic fungicides. However, the growing interest in organic farming and the concerns about toxic residues in food have prompted the search for more sustainable alternatives [10]. In this context, EOs derived from plants such as *Origanum vulgare*, *Salvia rosmarinus*, *Syzygium aromaticum*, *Thymus vulgaris*, and *Cinnamomum verum* have emerged as promising alternatives [11]. These oils not only possess antimicrobial properties, but have also been shown to extend the shelf life of fruits by providing fungitoxic effects and enhancing resistance to postharvest diseases [12,13].

EOs contain bioactive compounds such as terpenes and phenols, which are documented to have antimicrobial effects. These compounds can inhibit the growth of fungal pathogens, including those responsible for postharvest rot in bananas [14]. Previous studies have demonstrated the antifungal efficacy of EOs such as oregano, rosemary, and clove, although the results vary depending on the concentration, the type of fungus, and the storage conditions [15,16]. However, despite these promising results, there is limited research specifically evaluating the antifungal activity of these oils against *Fusarium* spp., *Penicillium* spp., *Trichoderma* spp., and *Aspergillus* spp. in bananas, especially under real postharvest conditions.

This investigation focuses on assessing the antifungal effectiveness of commercially available EOs against fungal pathogens isolated from banana peels. Existing research has helped in the development of sustainable alternatives to synthetic fungicides, promoting eco-friendly agricultural practices, and enhancing postharvest management [17]. Understanding the composition of the EOs used is crucial to identifying the bioactive compounds responsible for observed antifungal effects. The EOs of oregano and thyme share similar volatile compounds [18], such as the carvacrol, thymol, and p-cymene, and rosemary contains alpha-pinene, 1.8 cineol, camphor, and verbenone [19,20]. Clove oil is characterized by its content of eugenol, acetyl eugenol, and α and β caryophyllenes, and basil contains estragole, linalool, eugenol, and methyl cinnamate [19]. Cinnamon oil is rich in cinnamaldehyde and eugenol, which have demonstrated significant antifungal and bioactive properties [21].

Although EOs from oregano, basil, thyme, clove, cinnamon, and rosemary have a potent antifungal effects, their phytotoxicity must be carefully monitored. The excessive use or misdosage of these oils can cause damage to plants, such as chlorosis, necrosis, and reduced root growth, especially at concentrations above 1%. It is recommended to use safe concentrations of these oils and conduct preliminary tolerance tests on specific plant species before their large-scale application [11,22].

The objective of this study is to evaluate the potential of EOs as an ecological alternative for controlling fungal diseases in bananas, promoting sustainable agriculture and organic production. Specifically, this study evaluates the antifungal efficacy of these oils against *Trichoderma* spp., *Penicillium* spp., *Aspergillus* spp., and *Fusarium* spp. isolated from *Musa paradisiaca* through in vitro and ex vivo experiments.

*Fusarium* a filamentous genus known for its ability to adapt to diverse environmental conditions, is a major cause of vascular wilt in bananas, blocking the transport of water and nutrients, and reducing both the quality and quantity of fruit production [23]. In Ecuador is where one can generally find the species *oxysporum*, *verticillioides*, and *solani*, which are studied to innovate strategies to avoid damage to bananas [3,24].

*Penicillium* is distributed in various environments, and some species have antagonistic activity against pathogens that can cause deterioration of the fruit postharvest [25]. *P. citrinum*, *P. expansum*, and *P. digitatum* are frequently isolated from banana surfaces, and EOs like mandarin have been shown to inhibit their growth [26,27].

*Trichoderma* is a genus of filamentous, cosmopolitan fungi known for its biocontrol properties [28,29]. *Trichoderma* species produce antibiotics and secondary metabolites that inhibit phytopathogenic fungi [30]. India distributes certified species of *Trichoderma asperellum*, *T. atroviride*, *T. gamsii*, *T. hamatum*, *T. harzianum*, *T. polysporum*, *T. virens*, and *T. viride*, and with genetic engineering, has made significant improvements to their application in industrial processes [28,31].

*Aspergillus* spp. including species such as *A. niger*, *A. flavus*, and *A. parasiticus*, are common contaminants in bananas during storage and transportation [32]. These fungi produce a variety of enzymes that decompose plant tissues and can also produce harmful aflatoxins, which pose a risk to human health [14,32]. *Aspergillus parasiticus* is similar to *A. flavus*, and this species can also produce aflatoxin and affect the quality and safety of bananas [33].

In light of the above, the objective of the current study was to evaluate the potential of oregano, rosemary, clove, cinnamon, and basil EOs as an ecological alternative for controlling fungal diseases in bananas (*Musa paradisiaca*), promoting sustainable agriculture and organic production [34]. Specifically, this study evaluates the antifungal efficacy of these oils against *Trichoderma* spp., *Penicillium* spp., *Aspergillus* spp., and *Fusarium* spp. isolated from *Musa paradisiaca* through in vitro and ex vivo analysis. By promoting sustainable agriculture and reducing reliance on chemical fungicides, this research supports the development of more eco-friendly, effective strategies for postharvest disease management.

## 2. Materials and Methods

In this research, we used *Musa paradisiaca* (Ecuadorian bananas, which were harvested with export in mind). A sample of bananas was taken and exposed to an ambient temperature of approximately 25 °C until signs of rot were observed. Bananas that showed at least 50% of the symptoms of the presence of fungi in their peels were selected [35].

### 2.1. Isolation and Purification of Microorganisms

The isolation of pathogenic fungi from infected *Musa paradisiaca* peels was carried out using Potato Dextrose Agar (PDA) medium (Difco™, Detroit, MI, USA), widely recognized for its ability to promote fungal growth [36]. To inhibit bacterial contamination, chloramphenicol (Merck, Quito, Ecuador) was added at a final concentration of 0.5 g/L following the agar diffusion method [37]. The antibiotic was incorporated to prevent bacterial contamination during the fungal isolation process using the agar diffusion method. The prepared medium was then poured into sterile Petri dishes and allowed to solidify at room temperature.

The medium was prepared by dissolving 39 g of PDA in 1 L of distilled water, sterilized by autoclaving, poured into sterile Petri dishes, and left to solidify at room temperature [38]. Thirty banana samples with visible symptoms were collected, and approximately 20 g of peel was extracted per fruit. The peels were washed twice with sterile distilled water to remove superficial contaminants. Subsequently, the fragments were transferred to Erlenmeyer flasks containing 200 mL of a 0.05% (*v*/*v*) Tween 80 aqueous solution (Merck, Quito, Ecuador), and vortexed for 2 min to facilitate spore release.

Four serial dilutions (0.1%) were prepared from the suspension to ensure homogeneity [39]. From each dilution, 0.1 mL was inoculated onto PDA plates supplemented with 0.05% chloramphenicol and incubated at 25 ± 2 °C and 75 ± 5% relative humidity under a photoperiod of 12 h light/12 h dark [40]. Emerging colonies were monitored daily, individually isolated using a sterile loop, and subcultured weekly until pure cultures were obtained for macroscopic and microscopic morphological analyses [41].

### 2.2. Morphological Identification

Once pure fungi were obtained, macroscopic analysis was conducted to assess the characteristics of the fungal colonies, including their shape, color, and texture. For the identification of the causative agents affecting the banana, the upper and lower surfaces of the Petri dishes were observed macroscopically, considering the morphological similarities obtained through direct comparisons. The pure cultures were examined in triplicate weekly after inoculation with the isolated pathogens [42]. Their macroscopic characteristics were recorded and compared with bibliographic information from books and descriptive guides of fungal morphology to identify the genus of the pathogen [39,43]. Aspects such as the colony shape, elevation, edges and appearance of pure fungi were considered.

Microscopic examination was performed using conventional methods to observe the fungal reproductive structures, including hyphae, conidia, and spores. To visualize the microscopic and specialized structures, a piece of adhesive tape was used to collect the aerial mycelium of the fungus, and it was mounted on a microscope slide [39]. The plate was examined using a compound microscope (Motic Instruments Inc., Xiamen, China), equipped with a digital camera (Euromex Microscopes, Arnhem, The Netherlands), with 40× and 60× objective lenses, this analysis was performed in triplicate weekly. The evaluation was based on the observation of hyphae, mycelium, spores, and other microscopic structures present.

### 2.3. Molecular Identification Through DNA Sequencing

Molecular identification of fungi isolated from *Musa paradisiaca* peel was performed through DNA sequencing. Genomic DNA was extracted from pure fungal colonies using the commercial Invitrogen kit (Novogene, CA, USA), following the manufacturer’s instructions. The quality and quantity of the extracted DNA were evaluated using spectrophotometry with a Nanodrop spectrophotometer (Thermo Fisher Scientific, MA, USA) and by performing 1% agarose gel electrophoresis to assess DNA integrity [37].

The ribosomal DNA fragment corresponding to the Internal Transcribed Spacer (ITS) region was amplified using the universal primers ITS1 (5′-TCCGTAGGTGAACCTGCGG-3′) and ITS4 (5′-TCCTCCGCTTATTGATATGC-3′) [44]. The PCR amplification was carried out under optimal conditions in a thermocycler, and the amplified products were visualized on a 1.5% agarose gel stained with ethidium bromide under UV light to confirm successful amplification of the ITS region [37].

Purified amplicons were sent to Macrogen Inc. (Seoul, Republic of Korea) for sequencing. The obtained sequences were analyzed using BioEdit software, version 7.0 [45], for alignment and subsequent comparison with public databases, such as NCBI GenBank (https://www.ncbi.nlm.nih.gov/genbank/ (accessed on 10 April 2025)), using the BLASTn algorithm. The fungal species were identified based on a ≥98% similarity to deposited sequences in the database.

Phylogenetic analysis was conducted by aligning ITS sequences using the ClustalW algorithm. Phylogenetic trees were constructed employing both the Unweighted Pair Group Method with Arithmetic Mean (UPGMA) and the Neighbor-Joining approach, implemented in MEGA version 11 [46]. Pairwise, identity matrices and genetic distances were calculated based on the Tamura-Nei substitution model. The reliability of clade groupings was assessed through 1000 bootstrap replicates. This protocol is consistent with prior studies that support the effectiveness of ITS sequences for fungal identification and phylogenetic inference [11,47,48].

### 2.4. Ex Vivo Fungal Activity

After completing the macroscopic and microscopic characterization of the pathogens, the ex vivo antifungal activity was assessed. The pathogens were isolated and purified from banana peel samples and included *Trichoderma* spp., *Aspergillus* spp., *Penicillium* spp., and *Fusarium* spp. [49]. These fungi were selected from a total of 15 due to their relevance in postharvest diseases of bananas during storage.

The growth index was calculated based on the development of these fungi on fruit, with 20 samples used for each fungus and with four replicates for each treatment. The inoculum concentration was adjusted to 10^6^ conidia/mL to maintain constant infection [14]. A volume of 100 µL of the standardized fungal inoculum was applied, and the samples were maintained at approximately 13 ± 1 °C and 92 ± 3% relative humidity. The fungal growth diameter was measured weekly for up to 5 weeks post-inoculation, providing a comprehensive timeline of the pathogen’s progression and activity. The severity of infection was classified based on the growth diameter of the fungal colonies, with all of the purified pathogens considered in the analysis [43]. This classification provides valuable data on their interaction with the banana peel under controlled conditions [49]. A one-way ANOVA was applied to identify statistically significant differences in species growth, followed by Tukey’s HSD post hoc test.

### 2.5. In Vitro Antifungal Activity with Essential Oils

The antifungal efficacy of EOs derived from six species—oregano (*Origanum vulgare*), rosemary (*Salvia rosmarinus*), clove (*Syzygium aromaticum*), thyme (*Thymus vulgaris*), cinnamon (*Cinnamomum verum*), and basil (*Ocimum basilicum*)—was assessed in vitro using a method of incorporation into the medium [49,50,51]. Antifungal activity was assessed under controlled laboratory conditions at 25 ± 2 °C and 75 ± 5% relative humidity, with a photo period of 12 h of light and 12 h of darkness, and monitored daily. Essential oils (batch 20230516) supplied by Green Harmony were used, a company specialized in the production and distribution of pure and natural oils.

EOs were obtained using steam distillation, a widely accepted method for isolating plant-derived bioactive compounds. Oregano oil was extracted from dried leaves, cinnamon oil from dried bark, and clove oil from dried flower buds. For rosemary, basil, and thyme, EOs were derived from fresh leaves and flowers. All oils were purchased from certified commercial suppliers. Specifically, oregano EO originated from Turkey, cinnamon from Sri Lanka, clove from Indonesia, thyme from Japan, basil from India, and rosemary from Spain. It is worth noting that the geographic origin of EOs plays a critical role in determining their chemical composition and biological activity [52,53].

Cinnamon EOs consist mainly of cinnamaldehyde (60–70%) and eugenol (5–15%), both known for their antimicrobial and antioxidant effects. Basil EOs are characterized by their high contents of eugenol (50–70%), linalool (10–15%), and methyl chavicol (5–15%), which are compounds with antimicrobial, anti-inflammatory, and antioxidant properties. Oregano EOs are predominantly composed of carvacrol (60–80%), a phenolic compound recognized for its antimicrobial and antioxidant properties. It also contains thymol (5–10%), p-cymene (5–10%), and γ-terpinene (2–5%), which act together to inhibit microbial growth.

Clove EOs are distinguished by their high eugenol content (70–85%), followed by acetyl eugenol (5–15%) and β-caryophyllene (5–12%), all of which contribute to their antifungal and antibacterial effectiveness. The EOs of thyme contain thymol (30–50%) and carvacrol (10–20%), along with p-cymene (15–25%) and γ-terpinene (10–15%), which are responsible for their antifungal properties. The EOs of rosemary are mainly made up of 1,8-cineole (20–50%), carnosol (5–15%), rosmarinic acid (2–5%), and α-pinene (10–20%), compounds known for their antioxidant, antimicrobial, and potential neuroprotective effects.

To prepare the solutions, each EO was diluted in a 0.05% Tween 80 solution to form a homogeneous emulsion, at concentrations of 200, 400, 600, 800, and 1000 ppm [54]. These emulsions were added to cooled PDA medium before solidification. The pathogens were inoculated to determine the most effective concentration. Visual assessments were carried out every 24 h, considering three independent replicates and four subsamples per replicate for each microorganism. The mycelial growth inhibition percentages were calculated to assess the antifungal efficacy of the applied essential oils.

A negative control was included, consisting of PDA medium supplemented with 0.05% Tween 80 but without essential oils (EOs), allowing for the establishment of a baseline fungal growth reference in the absence of treatment. This facilitated the evaluation of the antifungal efficacy of the tested EOs. The experimental design followed a 6 × 5 mixed-factor model, where the independent variables were six different types of EOs and five concentration levels, while the dependent variable was the percentage of mycelial growth inhibition. Statistical analyses were performed to identify the optimal concentration of each EO capable of significantly inhibiting fungal growth. The findings provide meaningful insights into the potential of plant-derived essential oils as natural antifungal agents, supporting their future use in biocontrol strategies and sustainable agriculture.

## 3. Results

### 3.1. Morphological Identification

**Figure 1:**
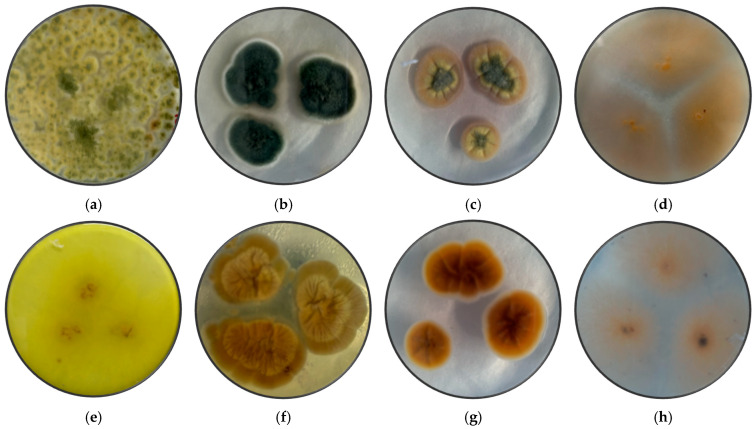
Macroscopic images of the front side of (**a**) *Trichoderma* spp, (**b**) *Penicillium* spp., (**c**) *Aspergillus* spp., and (**d**) *Fusarium* spp., and the reverse side of (**e**) *Trichoderma* spp., (**f**) *Penicillium* spp., (**g**) *Aspergillus* spp., (**h**) *Fusarium* spp.

**Figure 2:**
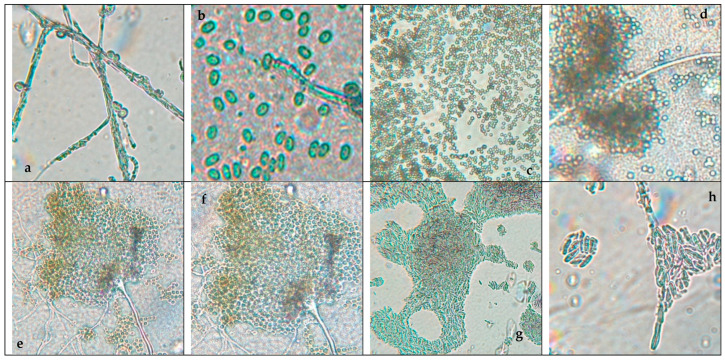
Microscopic images of (**a**) *Trichoderma* spp, (**c**) *Penicillium* spp., (**e**) *Aspergillus* spp., and (**g**) *Fusarium* spp. at 40× magnification and (**b**) *Trichoderma* spp., (**d**) *Penicillium* spp., (**f**) *Aspergillus* spp., and (**h**) *Fusarium* spp. at 60× magnification.

Figure 1 shows the pure fungi isolated from banana peel rot on selective medium (PDA + Chloramphenicol) and stored in PDA at approximately 25 °C, with macroscopic images of the front and reverse sides of *Trichoderma* spp., *Penicillium* spp., *Aspergillus* spp., and *Fusarium* spp. Figure 2 presents the aerial mycelium of the fungi. These observations provide visual information that complements the morphological analysis and microscopic images observed under 40× and 60× magnifications.

### 3.2. Molecular Identification Through DNA Sequencing

**Figure 3:**
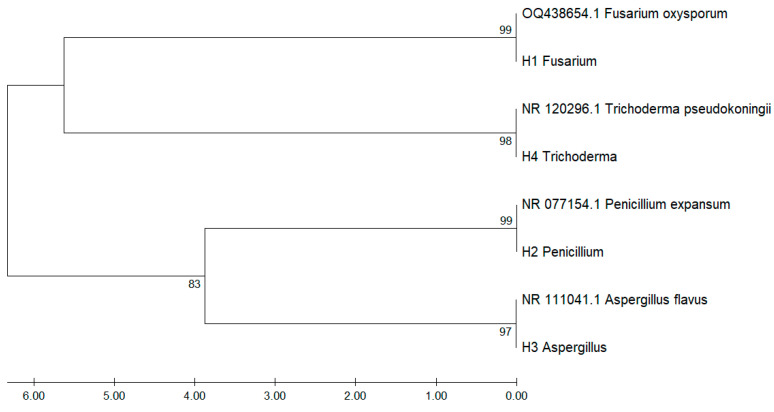
Phylogenetic tree based on ITS-region sequences of fungal isolates associated with *Musa paradisiaca*.

Figure 3 shows the phylogenetic tree constructed from ITS-region sequences of fungal isolates recovered from *Musa paradisiaca*, aligned against GenBank reference sequences. The analysis revealed consistent clustering by genus and species, supported by high bootstrap values confirming the molecular identification of the isolates.

Isolate H1 clustered closely with *Fusarium oxysporum* (OQ438654.1), with 99% bootstrap support, while H4 grouped with *Trichoderma pseudokoningii* (NR_120296.1) had 98% bootstrap support. Similarly, H2 aligned with *Penicillium expansum* (NR_077154.1) and H3 with *Aspergillus flavus* (NR_111041.1), with bootstrap values of 99% and 97%, respectively.

The phylogenetic tree also revealed two main clades: one comprising *Fusarium* and *Trichoderma*, and the other comprising *Penicillium* and *Aspergillus*, supported by a bootstrap value of 83%. These findings highlight the utility of ITS sequencing in accurately discriminating fungal pathogens in tropical crops such as banana.

Table 1 presents a summary of the results from sequencing the ITS region of fungi isolated from banana peels. It lists the identified fungal organisms, the genetic fragments utilized for sequencing, and the percentage similarity derived from a comparison with the database. The sequences exhibited a high degree of similarity (≥98%), which supports the reliability of the fungal genera and species identification in the analyzed samples.

The pairwise identity matrix derived from ITS sequence alignment (Table 2) revealed high similarity levels between the fungal isolates and GenBank reference sequences. Isolate H1 exhibited 99.6% identity with *Fusarium oxysporum* (OQ438654.1), while H4 showed 99.7% identity with *Trichoderma pseudokoningii* (NR_120296.1). Likewise, H2 matched *Penicillium expansum* (NR_077154.1) with 99.8% similarity, and H3 shared 99.2% identity with *Aspergillus flavus* (NR_111041.1).

The lowest identity values were observed between different genera, with genetic distances exceeding 10,000 units, highlighting clear taxonomic differentiation. This matrix quantitatively supports the phylogenetic proximity observed in the dendrogram and confirms molecular identification at the species level.

### 3.3. Fungal Activity Ex Vivo

**Figure 4:**
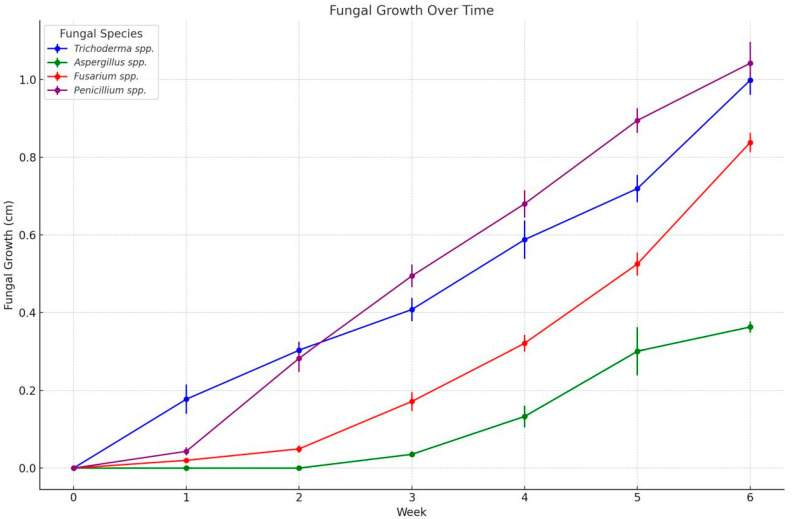
Fungal growth (cm) during 6 weeks in 20 banana samples inoculated with *Trichoderma* spp., *Penicillium* spp., *Aspergillus* spp., and *Fusarium* spp. stored at 13 °C and 95% HR approximately.

The severity of fungal infection in banana samples was assessed through ex vivo analysis, in which 20 banana samples were monitored over a 6-week period. Fungal growth was evaluated for various species, including *Trichoderma* spp., *Penicillium* spp., *Aspergillus* spp., and *Fusarium* spp., with Figure 4 providing a visual representation of this growth over time.

Regarding the progression of fungal growth on *Musa paradisiaca* peel samples over a six-week storage period, the analysis utilized an ANOVA approach to examine the impact of different treatments and concentrations on fungal development. Statistically significant differences were observed among treatments (*p* < 0.05), indicating that both the type of treatment and its concentration had a notable effect on fungal growth.

In addition, a one-way ANOVA revealed highly significant differences in growth among the four fungal species evaluated (*Penicillium*, *Trichoderma*, *Fusarium*, and *Aspergillus*) (F = 48.25, *p* < 0.001). These differences underscore the varying capacity of these fungi to colonize postharvest banana tissue.

Tukey’s Honest Significant Difference (HSD) post hoc test (α = 0.05) was conducted to identify pairwise differences. The results showed that *Penicillium* and *Trichoderma* exhibited the highest growth rates, with no significant difference between them (*p* > 0.05), while both displayed significantly greater growth compared to *Fusarium* and *Aspergillus*. Among all of the fungi, *Aspergillus* spp. showed the lowest growth, differing significantly from the other species (*p* < 0.001). These findings highlight the variability in fungal infections in bananas and emphasize the importance of effective treatment strategies to control postharvest decay.

Assumptions of normality and homogeneity of variances were verified through residual diagnostic plots and statistical tests. The Shapiro–Wilk test confirmed the normal distribution of residuals (*p* > 0.05), while Levene’s test indicated homogeneity of variances across groups (*p* > 0.05). Additionally, visual inspection of standard deviations revealed consistent dispersion among treatment groups, supporting the validity of the ANOVA results.

### 3.4. In Vitro Antifungal Activity with Essential Oils

**Figure 5:**
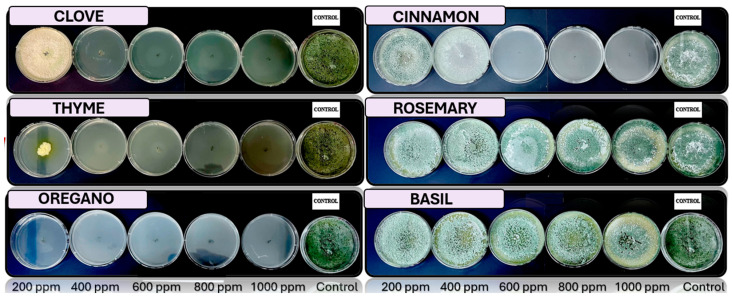
In vitro analysis of *Trichoderma* spp. in PDA medium supplied with basil, cinnamon, clove, oregano, rosemary, and thyme essential oils at various concentrations of 200, 400, 600, 800, and 1000 ppm (*n* = 12).

**Figure 6:**
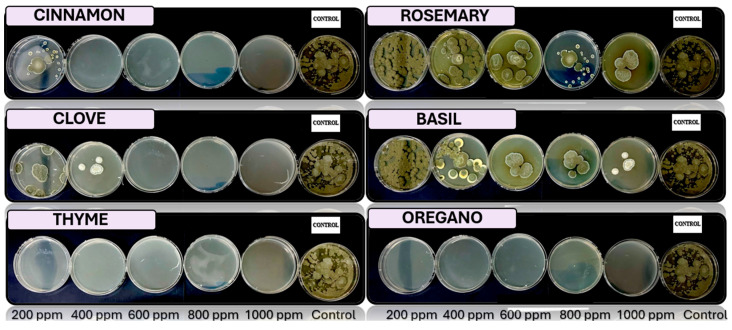
In vitro analysis of *Penicillium* spp. in PDA medium supplied with basil, cinnamon, clove, oregano, rosemary, and thyme essential oils at various concentrations of 200, 400, 600, 800, and 1000 ppm (*n* = 12).

**Figure 7:**
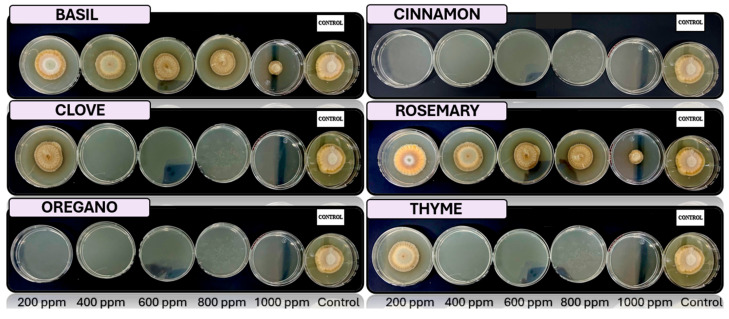
In vitro analysis of *Aspergillus* spp. in PDA medium supplied with basil, cinnamon, clove, oregano, rosemary, and thyme essential oils at various concentrations of 200, 400, 600, 800, and 1000 ppm (*n* = 12).

**Figure 8:**
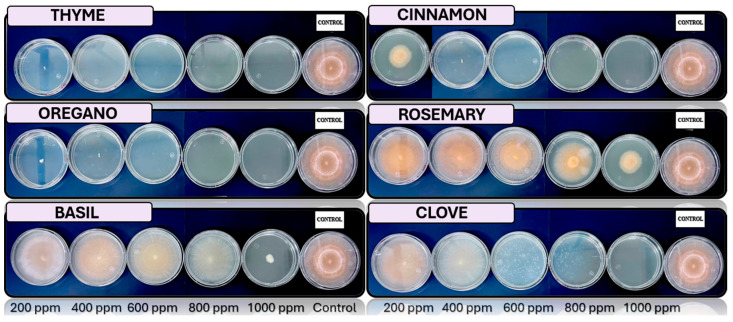
In vitro analysis of *Fusarium* spp. in PDA medium supplied with basil, cinnamon, clove, oregano, rosemary, and thyme essential oils at various concentrations of 200, 400, 600, 800, and 1000 ppm (*n* = 12).

Figure 5, Figure 6, Figure 7 and Figure 8 show the in vitro growth of *Trichoderma* spp., *Penicillium* spp., *Aspergillus* spp., and *Fusarium* spp., respectively, in PDA medium with 200 ppm, 400 ppm, 600 ppm, 800 ppm, and 1000 ppm of oregano, basil, cinnamon, rosemary, thyme, and clove EOs.

In this study, the antifungal efficacy of six essential oils (EOs) was evaluated in vitro against *Trichoderma* spp., *Penicillium* spp., *Aspergillus* spp., and *Fusarium* spp. at five concentration levels ranging from 200 to 1000 ppm. Fungal inhibition was recorded as the absence of visible mycelial growth and denoted by “−”, whereas growth was indicated by “+”. Table 3 presents a detailed overview of these inhibitory effects.

These results exhibit a clear concentration-dependent inhibition pattern. Cinnamon, oregano, and thyme oils demonstrated the highest antifungal efficacy, each achieving 23 out of 25 possible inhibitions across all tested fungi and concentrations. Conversely, basil oil displayed the weakest performance, with only 16 inhibitory responses.

Among the fungal genera, *Fusarium* spp. were the most sensitive, exhibiting consistent inhibition by most oils at concentrations of 400 ppm and above. *Trichoderma* spp. and *Penicillium* spp. showed moderate susceptibility, while *Aspergillus* spp. appeared comparatively more resistant, maintaining growth at lower concentrations for several oils.

These findings underscore the potential of cinnamon, oregano, and thyme essential oils as effective natural alternatives for the control of fungal pathogens in *Musa paradisiaca*, supporting their possible application in postharvest disease management strategies.

**Figure 9:**
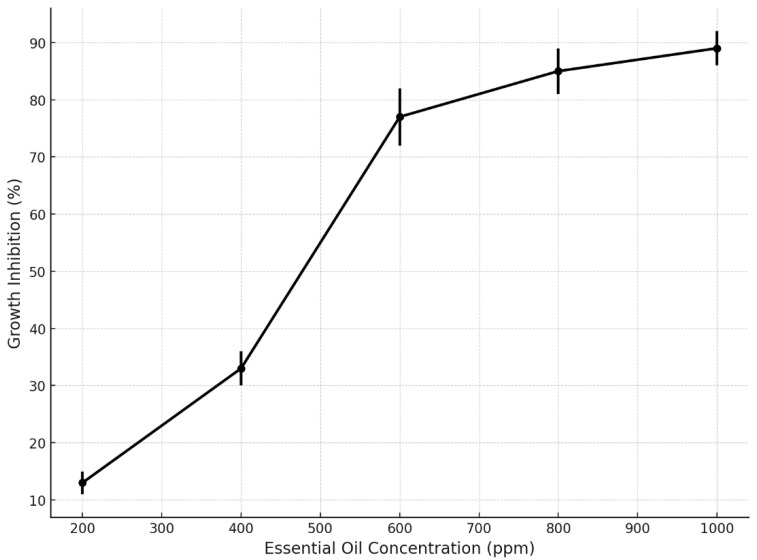
Fungal growth inhibition according to essential oil concentration.

The analysis of fungal growth inhibition revealed a clear concentration-dependent response to the application of essential oils (EOs). As shown in Figure 9, the inhibition percentages increased progressively with EO concentration, reaching statistically relevant thresholds. At the lowest tested concentration (200 ppm), the average inhibition was approximately 13%, while at 400 ppm it increased to 33%. A marked rise in antifungal activity was observed at 600 ppm, achieving 77% inhibition, with subsequent increases at 800 ppm (85%) and 1000 ppm (89%).

## 4. Discussion

### 4.1. Morphological Identification

In Figure 1 and Figure 2, the results of the macroscopic and microscopic analysis are presented and detailed.

*Trichoderma* spp. grow rapidly, with colonies that vary in color (white, green, yellow, or orange) and texture (cottony, velvety, or granular) depending on growth conditions. Microscopically, they produce branched conidiophores with small oval or cylindrical conidia that form in chains or clusters. A characteristic odor may be present but is not a reliable feature [55]. *Fusarium* spp. have networks of filaments (hyphae) and conidia (asexual and sexual spores or ascospores). Under a microscope, the phialides are generally thin, with a bottle shape that can be simple or branched and short or long, and they can have different characteristics depending on the species [56].

*Aspergillus* spp. are characterized by a prominent columella and highly organized plumose structures, where conidia emerge symmetrically. This distinct arrangement differentiates *Aspergillus* spp. from *Penicillium* and *Fusarium*, which have simpler conidiophore structures. *Penicillium* spp. can cause stains and discoloration on the peel of the banana, affecting its appearance and quality. *Expansum* can affect bananas during storage, causing rotting and loss of firmness, while *digitatum* is more common in citrus fruits but can affect bananas if they are stored in wet and warm storage conditions, causing rotting and stains on the peel [57]. To prevent the presence of this fungus, the fruit should be handled properly during storage and transport.

*Trichoderma* spp., including *T. asperellum*, *T. harzianum*, and *T. koningii*, are characterized by ellipsoidal conidia grouped together and thin, septate hyphae [58]. The colonies exhibit rapid growth and are typically green, with a circular shape, rough surface, regular edges, and slightly elevated contours [59]. The texture varies from fuzzy to cottony, reflecting their adaptability to different growth conditions.

*Penicillium* spp. display septate hyphae of variable length, forming structures that resemble plumes. The colonies range in color from white to green or blue, with a velvety surface and well-defined edges. The characteristic brush-like arrangement of conidia is a defining feature of this genus, enabling clear morphological differentiation from other fungi [25].

*Aspergillus* spp. are distinguished by septate hyphae and conidiophores bearing long, ellipsoidal conidia arranged in characteristic plumose structures. The colonies exhibit a range of colors, typically yellow to green, with a rugged surface and a texture resembling tufts. The edges are regular or slightly wavy, and the colony elevation often shows a cratiform pattern [33].

*Fusarium* spp. are characterized by fusiform, cylindrical conidia arranged in chains or clusters, supported by septate hyphae. The colonies appear pink, red, or orange, with a cottony or velvety texture, diffuse edges, and a flat or slightly elevated surface. These macroscopic and microscopic features distinguish *Fusarium* spp. from other genera [24].

This macroscopic and microscopic analysis of the fungal colonies, in combination with DNA sequencing, revealed distinct characteristics that facilitated the accurate identification of the species. The following discussion outlines the macroscopic features observed for *Fusarium oxysporum*, *Penicillium expansum*, *Trichoderma pseudokoringii*, and *Aspergillus flavus.*

*Fusarium oxysporum* exhibited typical characteristics of the *Fusarium* genus, including pink to orange colonies with a cottony texture and regular edges. The colony surface was flat or slightly elevated, which is consistent with the morphological description in [60]. The conidia, arranged in chains, were observable under the microscope. The macroscopic features, including the color and texture, provided reliable indicators for the identification of *Fusarium oxysporum* when compared to other species.

*Penicillium expansum* showed green colonies with white edges and a powdery, flat surface, which are characteristic of many *Penicillium* species, particularly those associated with decaying fruits and vegetables [61]. The white edge and powdery surface were key distinguishing features that aligned with the macroscopic description of *Penicillium expansum*. This appearance supports its identification, as it may have similar macroscopic features to other fungi, but differs in terms of details such as colony color or edge definition.

*Trichoderma pseudokoringii* include green colonies with a rough, granular surface and slightly elevated, regular edges. These features, common in many *Trichoderma* species [62], were consistent with the macroscopic description of *T. pseudokoringii*. The growing of the colonies and the distinctive texture helped confirm its identification. The rough surface and elevated edges serve as distinguishing features when compared to other fungi [30].

*Aspergillus flavus* exhibited yellow-green colonies with a rough surface and a cratiform elevation, consistent with the known morphological traits of *A. flavus* [33,63]. The coloration and rough texture of the colonies made it easy to differentiate *A. flavus* from other *Aspergillus* species. Its cratiform elevation, a distinct feature, further facilitated its identification. The yellow-green color and texture are particularly indicative of the species.

### 4.2. Molecular Identification Through DNA Sequencing

The DNA sequencing of fungi isolated from *Musa paradisiaca* samples provided highly reliable identification of the species through the ITS region, with all isolates showing a percentage identity above 98% [37]. These results validate the initial morphological analysis and emphasize the precision of molecular techniques in fungal taxonomy.

*Fusarium oxysporum* was identified with a 99.26% identity in the ITS region, confirming its classification within the *Fusarium* genus. This high similarity aligns with the macroscopic features previously observed, such as the pink-to-orange colonies with a cottony texture and regular edges. These combined findings provide strong evidence of its presence in the samples analyzed.

*Penicillium expansum* showed the highest percentage identity (99.68%) among the isolates, reflecting its genetic distinction. The molecular data corroborates its characteristic macroscopic traits, such as green colonies with white edges and a powdery, flat surface. The DNA sequencing and morphological analysis solidify its identification and highlight its significance in *Musa paradisiaca* samples and its association with fruit decay.

*Trichoderma pseudokoringii* exhibited a 99.24% identity, supporting its classification, which aligns with the observation of its rapid growth and green colonies with a granular texture. This genetic match enhances the confidence in its identification, particularly given the importance of *Trichoderma* species in biological control and agricultural systems.

*Aspergillus flavus* was identified with a 99.34% identity, confirming its classification. The sequencing results are consistent with their distinctive morphologies, such as yellow-green colonies with a rough surface and cratiform elevation. This high genetic identity underscores the reliability of ITS sequencing for distinguishing *A. flavus*, a species of economic and health significance due to its ability to produce aflatoxins.

The pairwise distance matrix derived from ITS sequences (Table 1 and Table 2) revealed significant genetic divergence among the fungal isolates analyzed. The lowest genetic distance was recorded between *Fusarium oxysporum* (OQ438654.1) and its corresponding local isolate, H1 (0.004), indicating a high degree of sequence similarity. In contrast, the highest divergence was observed between *Penicillium expansum* (NR_077154.1) and *Trichoderma pseudokoningii* (NR_120296.1), with values exceeding 16.4 units, reflecting distinct phylogenetic separation.

These findings align with the clustering patterns observed in the phylogenetic tree (Figure 3), further validating the internal transcribed spacer (ITS) region as a robust molecular marker for delineating taxonomic relationships among filamentous fungi. Moreover, the distance matrix (Table 2) reinforces the bootstrap-supported clade assignments, particularly between *Penicillium* and *Aspergillus* species, which exhibited moderate genetic distances.

### 4.3. Ex Vivo Fungal Activity

The inhibition rate was evaluated by measuring fungal growth from 20 inoculations of each fungus to assess the severity of the pathogens. The ex vivo growth of fungal species isolated from *Musa paradisiaca* was monitored over six weeks, as shown in Figure 4. The data demonstrates distinct growth patterns among the fungal species (*Trichoderma pseudokoringii*, *Aspergillus flavus*, *Fusarium oxysporum*, and *Penicillium expansum*), highlighting their varying rates of colonization and adaptation.

*Trichoderma pseudokoringii* exhibited the slowest growth among the species, with minimal increases in fungal colony size during the first three weeks. By week 6, the colony size had reached approximately 0.4 cm. The initial slow growth may reflect the competitive nature of *Trichoderma* spp. as a biological control agent, requiring time to adapt and establish in the substrate [62]. However, its accelerated growth in later weeks suggests its ability to utilize the available nutrients efficiently once adapted.

*Aspergillus flavus* displayed moderate growth, showing a consistent increase in colony size throughout the six weeks and reaching approximately 0.8 cm by the end of the observation period. This growth pattern aligns with its documented ability to colonize plant materials, particularly in nutrient-rich environments such as stored grains and fruits [64,65]. The steady growth of *A. flavus*’s indicates its adaptability and efficiency in utilizing *Musa paradisiaca* as a substrate.

*Fusarium oxysporum* demonstrated a growth rate between that of *Trichoderma pseudokoringii* and *Aspergillus flavus*. By week 6, the colony size was approximately 0.6 cm. The intermediate growth rate is consistent with the ecological behavior of *Fusarium* spp., which thrives under specific conditions but does not dominate rapidly in environments with competing organisms [55,60].

*Penicillium expansum* showed the highest growth rate among the species, reaching over 1 cm by week 6. This rapid growth reflects its opportunistic nature and strong ability to colonize decaying organic matter, including fruits like *Musa paradisiaca* [26,61,66]. A powdery texture and efficiency in nutrient utilization are characteristic of *P. expansum*, enabling its dominance in the substrate.

### 4.4. In Vitro Antifungal Activity with Essential Oils

The in vivo experiments with EOs at different concentrations (200, 400, 600, 800, and 1000 ppm) demonstrated distinct antifungal efficacy across fungal species (*Trichoderma pseudokoringii*, *Penicillium expansum*, *Aspergillus flavus*, and *Fusarium oxysporum*). These results emphasize the importance of EOs as natural antifungal agents in agricultural and food preservation applications.

Clove and cinnamon EOs contain active compounds such as eugenol and cinnamaldehyde, which have demonstrated significant antimicrobial properties. These compounds interact with fungal cell membranes, increasing their permeability and causing the leakage of essential components, which leads to fungal cell death [14].

Furthermore, cinnamaldehyde in cinnamon oil inhibits the production of intracellular enzymes such as amylases and proteases, resulting in the degradation of the cell wall and a high degree of cellular lysis. This action contributes to the efficacy of cinnamon oil in inhibiting the growth of various pathogens.

EOs also affect fungal spore germination. For instance, studies have shown that clove oil inhibits the germination of *F. oxysporum* and *F. solani* spores at concentrations of 125 µL/L, suggesting its potential as a preventive biofungicide. Likewise, thyme oil has been shown to inhibit the germination of *Colletotrichum acutatum* spores, significantly reducing the spread of this pathogen [67]. These findings support the efficacy of clove and cinnamon EOs as natural antimicrobial agents, offering sustainable alternatives for controlling fungal pathogens in the postharvest management of *Musa paradisiaca*.

*Trichoderma pseudokoringii* displayed a response to cinnamon and clove oils, which were most effective at higher concentrations (800–1000 ppm), showing significant inhibition of fungal growth, probably due to the phenolic compounds, such as cinnamaldehyde and eugenol, that disrupt fungal cell membranes [62]. Basil and rosemary oils exhibited moderate inhibition at all concentrations, while oregano and thyme oils showed minimal effects. These findings align with previous studies highlighting the potential of *Trichoderma pseudokoringii* as a biological control agent when combined with natural compounds [68].

The growth of *Penicillium expansum* was highly sensitive to cinnamon and clove oils, with complete inhibition observed at 800–1000 ppm. Basil and rosemary oils provided moderate inhibition across all concentrations, while thyme and oregano oils had limited effects. The pronounced sensitivity of *Penicillium expansum* to phenolic compounds suggests their potential use in controlling postharvest diseases in fruits and vegetables [69].

*Aspergillus flavus* showed significant growth inhibition when treated with cinnamon and clove oils, particularly at higher concentrations (800–1000 ppm). Thyme and oregano oils provided moderate inhibition, while basil and rosemary oils were less effective. The susceptibility of *Aspergillus flavus* to these oils underscores their utility in controlling fungal contamination and reducing aflatoxin production [69].

*Fusarium oxysporum* demonstrated varied responses to EOs. Cinnamon and clove oils showed moderate inhibition at concentrations above 600 ppm, while basil and rosemary oils exhibited consistent but limited effects across all concentrations. Oregano and thyme oils had minimal inhibitory activity. These results suggest that *Fusarium oxysporum* may require combined treatments, such as the integration of EOs with chemical fungicides or other biological agents, to achieve effective control [70].

Figure 9 shows an inhibition curve that reveals a clear concentration-dependent antifungal effect of essential oils, with inhibition rates increasing notably from 200 to 1000 ppm. A significant threshold was observed at 600 ppm, where the inhibition exceeded 75%, reaching nearly 90% at the highest concentration. These results highlight the effectiveness of EOs in fungal suppression, with reduced variability at higher doses, confirming their potential as natural antifungal agents for postharvest protection in *Musa paradisiaca*.

Cinnamon and clove oils exhibited the highest antifungal activity across all species, particularly at higher concentrations. Their effectiveness is attributed to their high phenolic content, which disrupts fungal cell structures and inhibits growth [71]. Basil and rosemary oils demonstrated moderate antifungal activity, suggesting their potential for combined use with other antifungal agents. Thyme and oregano oils exhibited limited antifungal effects [72,73], particularly against *Fusarium oxysporum* and *Trichoderma pseudokoringii*, highlighting the need for higher concentrations or combinations with other treatments.

## 5. Conclusions

Our findings showed that the characterization of fungi by order of severity identified *Penicillium expansum*, *Trichoderma pseudokoringii*, *Fusarium oxysporum*, and *Aspergillus flavus* as the fungi affecting *Musa paradisiaca* during the postharvest period. The in vitro analysis of essential oil efficacy revealed that *Penicillium expansum* was controlled by 400 ppm of cinnamon, oregano, and thyme essential oils. *Trichoderma pseudokoringii* was inhibited by 200 ppm of oregano, 400 ppm of clove and thyme, and 600 ppm of cinnamon essential oils. *Fusarium oxysporum* was most effectively managed with 200 ppm of cinnamon and thyme and 400 ppm of oregano essential oils. *Aspergillus flavus* was controlled by 200 ppm of oregano, and 400 ppm of cinnamon, clove, and thyme essential oils.

These findings suggest that essential oils, from cinnamon, clove, oregano, and thyme are promising natural alternatives to synthetic fungicides. They offer an eco-friendly solution for managing fungal infections in bananas. Future research should focus on optimizing their application in agricultural systems and investigating their synergistic effects with other control methods to enhance their antifungal efficacy

## Figures and Tables

**Table 1:** The sequencing results for fungi isolated from the samples of *Musa paradisiaca*.

Organism	Fragment	NCBI	% Identity
*Fusarium oxysporum*	ITS	OQ438654.1	99.26%
*Penicillium expansum*	ITS	NR_077154.1	99.68%
*Trichoderma pseudokoringii*	ITS	NR_111041.1	99.24%
*Aspergillus flavus*	ITS	NR_120296.1	99.34%

**Table 2:** Pairwise genetic distance matrix of ITS sequences among fungal isolates and GenBank reference strains.

	OQ438654.1	H1	NR_077154.1	H2	NR_111041.1	H3	NR_120296.1	H4
OQ438654.1	ID	0.004	14.540	14.540	15.618	15.609	15.323	15.361
H1	0.009	ID	14.553	14.553	15.681	15.676	15.310	15.349
NR_077154.1	12.564	12.512	ID	0.002	10.704	10.673	16.067	16.070
H2	12.564	12.512	0.004	ID	10.704	10.678	16.071	16.072
NR_111041.1	11.317	11.653	7.809	7.925	ID	0.004	14.602	14.562
H3	11.296	11.631	7.580	7.688	0.008	ID	14.555	14.530
NR_120296.1	11.091	11.091	16.486	16.336	9.969	10.412	ID	0.003
H4	11.396	11.396	16.445	16.295	10.425	10.044	0.007	ID

**Table 3:** Evaluation of in vitro antifungal activity of *Trichoderma* spp., *Penicillium* spp., *Aspergillus* spp., and *Fusarium* spp. upon using essential oils of oregano, rosemary, clove, thyme, cinnamon, and basil.

EOs	Fungus	Concentration [ppm]				
		200	400	600	800	1000
Cinnamon	*Trichoderma* spp.	+	+	−	−	−
	*Penicillium* spp.	+	−	−	−	−
	*Aspergillus* spp.	+	−	−	−	−
	*Fusarium* spp.	−	−	−	−	−
Clove	*Trichoderma* spp.	+	−	−	−	−
	*Penicillium* spp.	+	+	+	+	−
	*Aspergillus* spp.	+	−	−	−	−
	*Fusarium* spp.	+	+	−	−	−
Basil	*Trichoderma* spp.	+	+	+	+	+
	*Penicillium* spp.	+	+	+	+	+
	*Aspergillus* spp.	+	+	+	+	+
	*Fusarium* spp.	+	+	+	+	+
Oregano	*Trichoderma* spp.	−	−	−	−	−
	*Penicillium* spp.	+	−	−	−	−
	*Aspergillus* spp.	−	−	−	−	−
	*Fusarium* spp.	+	−	−	−	−
Rosemary	*Trichoderma* spp.	+	+	+	+	+
	*Penicillium* spp.	+	+	+	+	+
	*Aspergillus* spp.	+	+	+	+	+
	*Fusarium* spp.	+	+	+	+	+
Thyme	*Trichoderma* spp.	+	−	−	−	−
	*Penicillium* spp.	+	−	−	−	−
	*Aspergillus* spp.	+	−	−	−	−
	*Fusarium* spp.	−	−	−	−	−

## Data Availability

The original contributions presented in this study are included in the article. Further inquiries can be directed to the corresponding author.
